# Immunopathogenesis and immunoregulatory mechanisms in allergic contact dermatitis

**DOI:** 10.3389/fimmu.2026.1835083

**Published:** 2026-05-13

**Authors:** Yanjun Tong, Hong Chen, Qiong Kou, Bin He, Ying Tang, Kunjie Ran, Lijun Zhang, Xin Gan

**Affiliations:** 1 Department of Pharmacy, The First People’s Hospital of Guangyuan, Guangyuan, Sichuan, China; 2 Department of Dermatology, Ezhou Central Hospital, Ezhou, Hubei, China; 3 Department of Pharmacy, Chongren County Hospital of Traditional Chinese Medicine, Fuzhou, Jiangxi, China; 4 Department of Clinical Pharmacy, Dazhou Central Hospital, Dazhou, Sichuan, China; 5 Department of Pharmacy, Ezhou Central Hospital, Ezhou, Hubei, China

**Keywords:** allergic contact dermatitis, dendritic cells, immune response, inflammation, pattern recognition receptors, T cell

## Abstract

Allergic contact dermatitis (ACD) is a common inflammatory skin disorder characterized as a T cell-mediated delayed-type hypersensitivity reaction induced by cutaneous exposure to haptens. Its pathogenesis unfolds through three distinct phases: sensitization, elicitation, and resolution. During sensitization, hapten-modified proteins are processed by dendritic cells, particularly Langerhans cells and dermal dendritic cells, which migrate to lymph nodes to prime naive T cells. Pattern recognition receptors, including Toll-like receptors and the NLRP3 inflammasome, critically regulate this innate-adaptive interface. The elicitation phase involves hapten-specific Th1 and Th17 cells orchestrating inflammation through cytotoxicity and cytokine release. Conversely, resolution relies on regulatory T cells and IL-10 and TGF-beta signaling to restore tissue homeostasis. Emerging immunomodulators such as vitamin D exhibit dose-dependent regulatory effects potentially influenced by sex-specific factors and U-shaped associations. Despite significant advances, critical gaps persist regarding tissue-resident memory T cells and precise sensitization thresholds. Integrating immunology, neurobiology, and metabolomics may advance precision therapies targeting pathways like NLRP12 or ultraviolet-induced vitamin D synthesis. This review summarizes current progress in elucidating the immunopathogenesis of ACD and highlights emerging mechanisms that may support the development of more precise and effective therapeutic strategies.

## 1 Introduction

Allergic contact dermatitis (ACD) represents a prevalent T cell–mediated delayed-type hypersensitivity disorder, affecting 15–20% of individuals in industrialized regions and imposing substantial clinical and socioeconomic burdens (1). Pathogenetically, ACD is triggered by cutaneous exposure to low-molecular-weight haptens that covalently modify endogenous proteins, generating immunogenic neoantigens (2). During sensitization, hapten-modified keratinocytes release alarmins that activate dendritic cells (DCs), which migrate to draining lymph nodes to prime naïve T cells (3, 4). Upon re-exposure, hapten-specific effector T cells orchestrate local inflammation via cytotoxicity and proinflammatory cytokine release, whereas resolution relies on regulatory T cells and immunosuppressive cytokines to restore tissue homeostasis (5, 6).

Notwithstanding advances in delineating canonical DC–T cell axes, critical gaps persist regarding the molecular determinants of sensitization thresholds, the functional heterogeneity of tissue-resident memory T cells in disease recurrence, and the context-dependent roles of pattern recognition receptors beyond inflammasome signaling (7–10). Recent investigations increasingly implicate neuroimmune crosstalk, metabolic reprogramming, and immunomodulatory axes as pivotal determinants orchestrating ACD pathogenesis (11, 12). This review summarizes the current insights into the cellular networks and molecular checkpoints orchestrating each phase of ACD, with particular emphasis on emerging mechanisms—including NLRP12-mediated negative regulation and ultraviolet radiation-induced immunomodulation—that hold translational promise for advancing precision therapeutic strategies against this complex inflammatory dermatosis.

## 2 Sensitization phase of allergic contact dermatitis

### 2.1 Dose-dependent tolerance mechanisms in allergic contact dermatitis

The immunogenicity of contact allergens exhibits a non-linear, dose-dependent relationship, wherein supraphysiological exposure levels can paradoxically attenuate sensitization responses relative to concentrations that optimally prime adaptive immunity (13). This biphasic phenomenon, often conceptualized within the framework of high-zone tolerance, is critically modulated by the anatomical route of allergen entry. Gastrointestinal exposure to elevated hapten doses induces functional anergy in antigen-reactive T lymphocytes, thereby abrogating secondary recall responses (14). Intravenous administration preferentially expands regulatory T cell (Treg) populations, leading to active suppression of effector T cell function (15). In contrast, epicutaneous application of high-dose haptens impairs dendritic cell migration from the epidermis to draining lymph nodes, consequently disrupting the afferent limb of sensitization (16). These route-specific tolerogenic mechanisms offer conceptual foundations for prophylactic interventions aimed at preventing ACD onset. Nevertheless, quantitative thresholds delineating sensitizing versus tolerogenic exposure regimens remain ill-defined across diverse hapten classes. Rigorous dose-response characterization, integrated with kinetic profiling of innate immune activation, is essential to translate these mechanistic insights into clinically actionable strategies for risk assessment and preventive dermatology.

### 2.2 Dendritic cell subsets and antigen presentation in allergic contact dermatitis

Dendritic cells function as the principal antigen-presenting cells (APCs) bridging innate and adaptive immunity within the cutaneous microenvironment. Skin-resident DCs are compartmentalized into epidermal Langerhans cells (LCs) and heterogeneous dermal dendritic cell (DDC) subsets (17, 18). Upon hapten exposure, keratinocytes act as immunological sentinels, releasing IL-1β that signals through IL-1RI on resident DCs to initiate maturation and local TNF-α production (9, 19). Keratinocytes release IL-1β upon antigenic challenge, which activates DCs via IL-1RI, prompting TNF-α production (20, 21). TNF-α signaling through TNF-RII enhances DC detachment by downregulating E-cadherin (CD324) and promoting MMP-mediated degradation of type IV collagen (19, 22). Successful egress to draining lymph nodes is governed by dynamic chemokine receptor reprogramming. Antigen encounter downregulates inflammatory receptors (CCR1, CCR5, CCR6) while upregulating lymphoid-homing receptors (CCR7, CXCR4, CCR4) to guide directional trafficking (19, 23). This migratory switch is amplified by lipid mediators such as cysteinyl leukotrienes and prostaglandin E2, whereas IL-4, IL-10, and TGF-β1 exert suppressive effects (24–26). DDCs exhibit rapid migratory kinetics due to their strategic dermal positioning, whereas LCs require extended detachment periods and pronounced CCR7 upregulation before lymphatic entry. Functionally, LCs preferentially cross-present hapten-modified antigens to prime CD8^+^ cytotoxic T lymphocytes (CTLs), while DDCs efficiently activate CD4^+^ T helper cells through IL-12 and IL-23 secretion, driving Th1 and Th17 polarization (27).

Notably, specific DDC subsets also contribute to peripheral tolerance by producing IL-10 or engaging regulatory T cell pools (28). The maturation and functional programming of DCs are tightly coordinated by pattern recognition receptor (PRR) sensing and downstream MAPK signaling. Hapten-protein conjugates and endogenous danger ligands engage surface and intracellular PRRs, including Toll-like receptors (TLR1, TLR2, TLR4, TLR5, TLR6, TLR7) and C-type lectins such as CD209 (29). PRR engagement converges on MAPK cascades: inflammatory and stress stimuli activate JNK and p38 pathways, whereas receptor tyrosine kinase signaling stimulates ERK (30, 31). Among these, the p38 MAPK axis functions as a master regulator, orchestrating the transcriptional upregulation of essential co-stimulatory molecules (CD40, CD80, CD83, CD86) and MHC class II complexes (32, 33). This integrated signaling architecture ensures that DCs acquire the requisite immunostimulatory capacity to effectively prime naïve T cells, while its precise modulation dictates the balance between sensitization and tolerance.

### 2.3 Pattern recognition receptors in immune surveillance

PRRs function as critical molecular sentinels that translate cutaneous danger signals into coordinated innate and adaptive immune responses in ACD (34). Although evolutionarily optimized for microbial pathogen-associated molecular patterns (PAMPs), PRRs in contact hypersensitivity primarily recognize exogenous haptens and endogenous damage-associated molecular patterns (DAMPs) released upon epidermal barrier disruption (35, 36). This response relies on PRRs, which detect pathogen-associated molecular patterns (PAMPs) from microorganisms. PRRs are expressed in various cell types, including macrophages, DCs, neutrophils, and keratinocytes. The PRR family includes TLRs, nucleotide-binding oligomerization domain-like receptors (NLRs), CLRs, and retinoic acid-inducible gene I-like receptors (RLRs), with TLRs and NLRs being the main mediators (37). During sensitization, metal allergens like nickel activate TLR4 on keratinocytes, inducing cytokine release and DC migration to lymph nodes, facilitating T cell priming (38, 39). NLRs further modulate the inflammatory landscape through inflammasome-dependent and independent mechanisms.

Epidermal injury and hapten-induced oxidative stress release DAMPs such as ATP and reactive oxygen species (ROS), which activate the NLRP3 inflammasome in myeloid and epithelial compartments (40, 41). This multiprotein complex promotes caspase 1–mediated proteolytic maturation of pro-IL-1β and pro-IL-18, skewing the local cytokine milieu toward a Th1-polarized phenotype. In sharp contrast, NLRP12 functions as a potent endogenous brake on contact hypersensitivity. Silencing of Blimp-1, a transcriptional repressor of NLRP12, derepresses NLRP12 expression and concurrently suppresses IL-18 production, establishing NLRP12 as a pre-exposure checkpoint that constrains exaggerated immune activation (42). The kinetics of the Blimp-1/NLRP12 axis are finely tuned by hapten potency; strong sensitizers such as dinitrochlorobenzene (DNCB) rapidly induce Blimp-1 and downstream IL-18 release, whereas weaker allergens like para-phenylenediamine elicit delayed and attenuated responses (43). CLRs additionally contribute to sterile inflammation by sensing lipid and carbohydrate moieties liberated from damaged keratinocytes. Cutaneous trauma upregulates Mincle (Clec4e), which recognizes cholesterol sulfate released from necrotic epidermal cells (44). Mincle engagement activates Syk-CARD9 signaling, amplifying dendritic cell activation and driving robust Th17 cell expansion, thereby exacerbating tissue inflammation (45) (**Table 1**).

**Table 1:** Summary of key pattern recognition receptors in allergic contact dermatitis pathogenesis.

PRR family	Receptors	Recognized ligands	Signaling pathways	Cellular sources	Inflammatory factors	Function
Toll-like receptors (TLRs)	TLR2, TLR3, TLR4, TLR7, TLR9	Bacterial lipopeptides (TLR2), dsRNA (TLR3), nickel/cobalt ions (TLR4), ssRNA (TLR7), unmethylated CpG DNA (TLR9)	MyD88/NF-κB, TRIF/IRF3	Keratinocytes, DCs, macrophages, Langerhans cells	IL-1β, TNF-α, IL-6, type I IFNs; CCR7 on DCs	Initiates DC maturation, promotes skin inflammation, triggers T cell priming via enhanced antigen presentation
NOD-like receptors (NLRs)	NLRP3, NLRP12, NLRC4	DAMPs: extracellular ATP, ROS, uric acid crystals, bacterial peptidoglycans	IL-1β, IL-18; NLRP12: NF-κB repression	Keratinocytes, DCs, monocytes	IL-1β, IL-18 (NLRP3); anti-inflammatory tone (NLRP12)	Amplifies inflammation (NLRP3); promotes Th1/Th17 skewing; NLRP12 acts as an inflammation brake
C-type lectin receptors (CLRs)	Mincle (Clec4e), Dectin-1, Dectin-2	Cholesterol sulfate (Mincle), fungal β-glucans (Dectin-1)	NF-κB, MAPKs	Dermal DCs, macrophages	IL-6, IL-23, TNF-α; promotes Th17 differentiation	Links epidermal injury to Th17 polarization; promotes DC activation and local inflammation
RIG-I–like receptors (RLRs)	RIG-I, MDA5	Cytosolic viral RNA (short/long dsRNA)	IRF3/7, NF-κB	DCs, keratinocytes	Type I IFNs (IFN-α/β), ISGs	Not well defined in ACD; may amplify inflammation during viral-induced flares
Absent in melanoma 2 (AIM2)-like receptors (ALRs)	AIM2	Cytosolic dsDNA	IL-1β, IL-18	Keratinocytes, DCs	Inflammasome-dependent cytokines	Potential amplification of inflammation in hapten-induced injury
Scavenger receptors (SRs)	SR-A1, MARCO	Oxidized LDL, apoptotic cell debris	Src kinase, MAPKs	Macrophages, DCs	Phagocytosis, ROS	Aid in clearance of haptens/apoptotic keratinocytes; modulate inflammation

### 2.4 Immune cell populations in allergic contact dermatitis

The initial phase of ACD is rapidly orchestrated by a coordinated influx of innate and innate-like immune populations that establish the inflammatory and metabolic milieu prerequisite for adaptive priming. Tissue-resident macrophages and recruited monocytes accumulate at the hapten-challenged site within 24–48 hours, serving as critical signaling and regulatory hubs (46). Their functional polarization is governed by the arginase-1 (Arg1)–inducible nitric oxide synthase (iNOS) metabolic axis; exposure to potent sensitizers such as DNBS preferentially upregulates iNOS while suppressing Arg1, thereby skewing macrophages toward a proinflammatory phenotype that exacerbates disease severity (47). Conversely, early Arg1 dominance can mitigate inflammation through substrate competition and tissue repair signaling. Neutrophils exhibit even more rapid kinetics, peaking within 24 hours of epidermal challenge (48, 49). Although traditionally characterized as purely destructive, neutrophils display context-dependent plasticity, acquiring regulatory functions via IL-10 secretion or modulating neutrophil extracellular trap (NET) dynamics during later disease stages (50).

Innate-like lymphocytes further calibrate the threshold for adaptive immune activation. Natural killer T (NKT) cells recognize lipid-hapten complexes, particularly in metal-induced hypersensitivity (nickel), where they license dendritic cells and secrete IFN-γ to drive early Th1 polarization (51–53). Epidermal γδ T cells act as rapid sentinels, releasing IL-17A in response to keratinocyte-derived stress signals to reinforce barrier integrity and recruit myeloid effectors (54). By promoting dendritic cell maturation and amplifying localized inflammatory cascades, γδ T cells effectively bridge innate detection and adaptive priming (55, 56). Mast cells, strategically positioned at barrier interfaces, amplify early vascular permeability and leukocyte recruitment through the release of histamine, proteases, and cytokines (IL-3, IL-4, IL-6) upon hapten or danger-signal recognition (57). However, they exhibit biphasic functionality, transitioning to IL-10 production during the resolution phase to constrain tissue damage (58). NK cells, comprising approximately 10% of the early lymphocytic infiltrate, are recruited by Th1/Th17-derived cytokines (59). Human NK cells exhibit functional heterogeneity, encompassing lymph node-resident immunoregulatory subsets and peripheral tissue-homing memory-like populations (60, 61). During elicitation, memory-like NK cells mount rapid recall responses, sustaining local inflammation through IFN-γ and cytotoxic mediator release for weeks to months, underscoring the potential contribution of NK cells to long-term cutaneous immune memory (62).

## 3 Activation phase of allergic contact dermatitis

### 3.1 T cell activation

The priming of naïve T lymphocytes is governed by a strictly regulated three-signal paradigm that integrates antigen recognition, co-stimulation, and cytokine-directed programming. Signal 1 originates from the cognate engagement between the T cell receptor (TCR) and peptide–major histocompatibility complex (pMHC) assemblies on dendritic cells (63, 64). Exogenous haptens are typically internalized, processed within endolysosomal compartments, and presented via MHC class II molecules to CD4^+^ T cells. Conversely, cytosolic antigens undergo proteasomal degradation and load onto MHC class I molecules for CD8^+^ T cell recognition. Notably, cross-presentation enables certain contact sensitizers and exogenous neoantigens to access the MHC class I pathway, significantly expanding the cytotoxic T cell repertoire in contact hypersensitivity (65). The secondary signal is mediated by co-stimulatory ligands expressed on the surface of APCs, specifically CD80, CD40, and CD86. This secondary signal sustains TCR signaling cascades, promotes IL-2 autocrine production, and prevents activation-induced cell death. Concurrently, the inhibitory receptor CTLA-4 (CD152) competitively engages B7 ligands with superior affinity, delivering a critical checkpoint that constrains excessive T cell expansion and maintains peripheral tolerance (63). Subsequently, DCs secrete a specific cytokine milieu that constitutes the third critical signal, directing T cell polarization. In ACD, DC-derived IL-12 and IL-23 drive Th1 and Th17 differentiation, characterized by robust IFN-γ, TNF-α, and IL-17 production, respectively. While IL-4-rich environments favor Th2 polarization, its pathogenic contribution to ACD remains context-dependent and frequently overshadowed by Th1/Th17 dominance. The absence of co-stimulatory or cytokine signals typically induces clonal anergy or apoptosis, establishing a vital safeguard against aberrant immune activation (66, 67). Following antigen clearance, surviving T cells undergo terminal differentiation into functionally specialized memory subsets. Effector memory T cells (Tem) downregulate CCR7, enabling rapid extravasation into inflamed peripheral tissues and immediate execution of cytotoxic and inflammatory programs upon rechallenge. In contrast, central memory T cells (Tcm) retain CCR7 expression, facilitating continuous recirculation between the circulation and secondary lymphoid organs. This strategic compartmentalization ensures both immediate cutaneous surveillance and long-term systemic immunological readiness, underpinning the recurrent nature of ACD upon hapten re-exposure (68, 69).

### 3.2 T cell effector functions

The elicitation phase of ACD is predominantly driven by the coordinated effector functions of CD4^+^ and CD8^+^ T lymphocyte subsets. Among CD4^+^ lineages, Th1 and Th17 cells serve as the principal orchestrators of cutaneous inflammation and tissue injury. Th1-derived IFN-γ and TNF-α activate keratinocytes, upregulate adhesion molecules, and recruit macrophages, while Th17-secreted IL-17A and IL-22 synergize to amplify neutrophil infiltration, compromise epidermal barrier integrity, and perpetuate inflammatory amplification (70). Among CD4^+^ lineages, the Th1 and Th17 subsets have been implicated as principal drivers of tissue injury and inflammatory amplification (71). The pathogenic contribution of Th2 cells remains highly context-dependent; although traditionally linked to atopic pathology, accumulating evidence indicates they may exert either immunoregulatory or pro-inflammatory effects in ACD, largely dictated by hapten chemical properties, exposure kinetics, and the local cytokine milieu (67, 72). CD8^+^ cytotoxic T lymphocytes constitute the dominant effector population responsible for direct keratinocyte damage and lesion propagation. Their cytolytic activity is executed through two mechanistically distinct, non-redundant pathways. The primary route involves calcium-dependent granule exocytosis, wherein perforin polymerizes to form transmembrane pores, facilitating the targeted delivery of granzymes into the cytosol. Granzyme B subsequently cleaves executioner caspases and the pro-apoptotic protein Bid, triggering rapid, receptor-independent apoptosis (73). The secondary pathway operates via death receptor signaling, wherein CTL surface-expressed Fas ligand (FasL/CD95L) engages constitutively expressed Fas (CD95) on target keratinocytes. This engagement nucleates the death-inducing signaling complex (DISC), culminating in caspase-8 activation and downstream apoptotic cascades (70). Notably, experimental models of contact hypersensitivity demonstrate strict functional non-redundancy between these axes; genetic or pharmacological ablation of either the perforin/granzyme or Fas/FasL pathway significantly attenuates, but does not fully abolish, tissue injury, underscoring their complementary roles in disease pathogenesis (65).

## 4 Immunoregulatory mechanisms in allergic contact dermatitis

**Figure 1:**
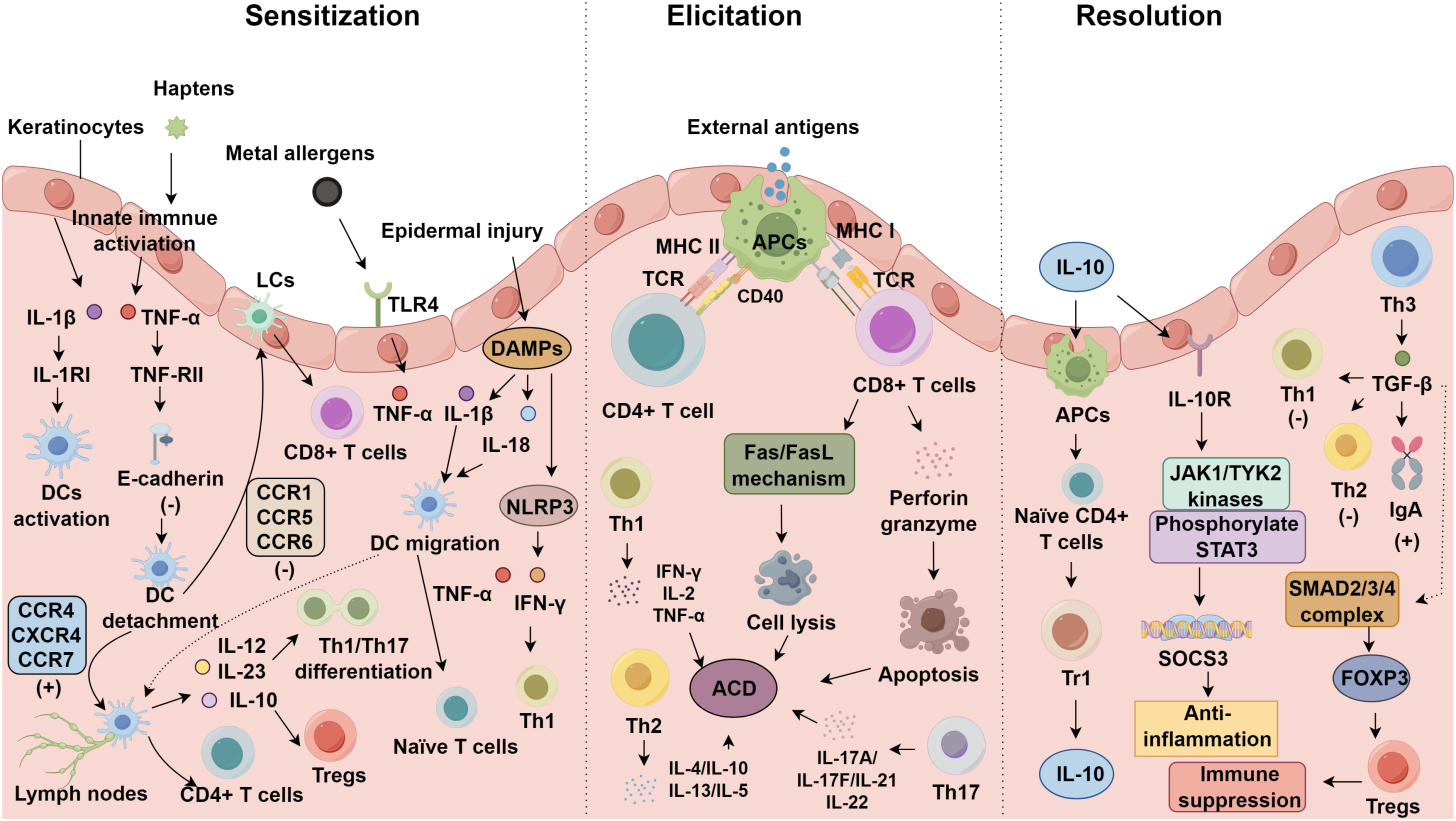
Immunological mechanisms of allergic contact dermatitis.

The balance between effector T lymphocyte activity and Treg function is a critical factor in ACD progression (74). T lymphocytes are classified into three subsets based on their cytokine profiles: Tr1 cells, which primarily secrete IL-10; Th3 cells, which produce TGF-β; and CD4^+^CD25^+^ T cells (75). In IL-10–rich microenvironments, antigen-presenting cells promote the differentiation of naïve CD4^+^ T cells into Tr1 cells via STAT3-dependent signaling. IL-10 engagement of its heterodimeric receptor activates JAK1 and TYK2 kinases, triggering STAT3 phosphorylation, nuclear translocation, and transcriptional induction of anti-inflammatory mediators such as SOCS3, which attenuates NF-κB and MAPK signaling cascades (76, 77). IL-10 production peaks within 10–14 hours post-activation and exerts potent immunomodulatory effects during the initial 24 hours of the response, with mast cells and macrophages serving as auxiliary sources (78–80). Th3 cells, defined by TGF-β secretion, contribute to mucosal tolerance by promoting IgA class switching and suppressing Th1/Th2 polarization (81, 82). TGF-β signaling is indispensable for the peripheral induction of CD4^+^CD25^+^FOXP3^+^ Tregs. Upon ligand binding, the type II TGF-β receptor recruits and phosphorylates the type I receptor, which subsequently phosphorylates SMAD2 and SMAD3 transcription factors. These activated SMAD proteins associate with SMAD4, forming complexes that translocate to the nucleus to initiate FOXP3 expression. This signaling axis not only fosters peripheral immune tolerance but also actively suppresses the activation and proliferative expansion of effector T cells (83). Beyond cytokine-mediated suppression, CD4^+^CD25^+^FOXP3^+^ Tregs enforce tolerance through direct cell–cell interactions, including CTLA-4–dependent downregulation of CD80/CD86 on dendritic cells, lymphocyte activation gene-3 (LAG-3)–mediated inhibition of antigen presentation, and metabolic disruption via CD39/CD73 ectoenzyme activity that converts proinflammatory ATP to immunosuppressive adenosine (84). The maturation status of antigen-presenting cells further shapes the effector–regulatory balance: immature or tolerogenic DCs favor Treg expansion, whereas fully matured DCs promote effector differentiation (85). Complementing Treg activity, regulatory B cells (Bregs)—typically identified by CD1d^+^CD5^+^ surface markers and defined functionally by IL-10 secretion—constitute an additional immunomodulatory axis that constrains contact hypersensitivity and facilitates resolution (86, 87) (**Figure 1**).

## 5 The immunomodulatory role of vitamin D in allergic contact dermatitis

The vitamin D receptor (VDR) is widely expressed across immune cell types, including dendritic cells, macrophages, T and B lymphocytes, and neutrophils, enabling direct transcriptional regulation of genes governing inflammation, antimicrobial defense, and cellular differentiation vitamin D signaling attenuates proinflammatory cascades by downregulating. Toll-like receptor expression in monocytic lineages, suppressing lipopolysaccharide-induced dendritic cell maturation, and inhibiting Th1/Th17 polarization while promoting regulatory T cell differentiation (88). Additionally, vitamin D enhances autophagic flux through mTOR pathway inhibition and induces cathelicidin antimicrobial peptide transcription, thereby limiting pyroptosis-associated tissue damage and mitigating sterile inflammation (88, 89). Clinical and preclinical evidence regarding vitamin D in inflammatory dermatoses reveals considerable complexity. While some observational studies report an inverse correlation between circulating 25-hydroxyvitamin D levels and disease severity in atopic dermatitis (90), broader literature yields inconsistent associations, with certain cohorts suggesting that elevated vitamin D status may paradoxically increase allergic susceptibility. Sex-dimorphic responses further complicate interpretation: vitamin D deficiency exacerbates contact hypersensitivity in male mice but exerts minimal effects in females, hinting at hormonal or genetic modifiers of VDR signaling (91) (92). Hyppönen et al. (93) identified a U-shaped association between serum 25-hydroxyvitamin D and immunoglobulin E levels, implying that immune homeostasis requires vitamin D concentrations within a narrow physiological window. Future investigations should prioritize well-powered prospective cohort studies and randomized controlled trials to delineate causal relationships between vitamin D status and ACD outcomes. Such efforts may ultimately inform personalized supplementation strategies that leverage ultraviolet radiation–induced cutaneous vitamin D synthesis as a tolerogenic adjunct in the management of contact hypersensitivity.

## 6 Conclusion

The pathophysiological architecture of ACD is predicated upon a sophisticated, dynamic reciprocity between innate and adaptive immune compartments, orchestrated by hapten-specific T lymphocyte clones and finely tuned by endogenous immunosuppressive circuitry. Contemporary investigations have elucidated pivotal determinants governing the sensitization phase, revealing that allergen potency operates in a dose-dependent manner, while functional heterogeneity among dendritic cell subsets and the engagement of specific PRRs—notably TLR4 and the NLRP3 inflammasome—serve as critical gatekeepers of immune initiation. Subsequently, the elicitation phase is predominantly driven by cytotoxic cascades emanating from Th1 and Th17 effector lineages, whereas the resolution of inflammation hinges upon Treg-mediated signaling axes involving interleukin-10 (IL-10) and TGF-β. Furthermore, emerging conceptual frameworks emphasizing neuroimmune integration—exemplified by substance P-induced mast cell degranulation—and metabolic modulation, including vitamin D homeostasis and the Arg1–iNOS regulatory axis, provide fertile ground for the development of novel therapeutic modalities.

Notwithstanding these significant strides, substantial knowledge gaps persist regarding the precise molecular determinants dictating immune sensitization thresholds, the contributory role of tissue-resident memory T cells in disease recurrence, and the nuanced modulatory capacity of diverse PRRs beyond current characterization. Potential therapeutic horizons may involve the selective targeting of negative regulators such as NLRP12 or the exploitation of ultraviolet radiation (UVR)-induced cutaneous vitamin D synthesis to restore immune tolerance. Ultimately, transcending current limitations necessitates a convergent, multidisciplinary paradigm that synthesizes insights from immunology, neurobiology, and metabolomics, thereby paving the way for precision medicine strategies tailored to the complex etiology of ACD.

## References

[1] Brites GS Ferreira I Sebastião AI Silva A Carrascal M Neves BM . Allergic contact dermatitis: from pathophysiology to development of new preventive strategies. Pharmacol Res. (2020) 162:105282. doi: 10.1016/j.phrs.2020.105282. PMID: 33161140

[2] Jurakić Tončić R Lipozenčić J Martinac I Gregurić SJADC . Immunology of allergic contact dermatitis. (2011) 19:51–68. 21489368

[3] Peiser M Tralau T Heidler J Api AM Arts JH Basketter DA . Allergic contact dermatitis: epidemiology, molecular mechanisms, *in vitro* methods and regulatory aspects. Current knowledge assembled at an international workshop at BfR, Germany. Cell Mol Life Sci. (2012) 69:763–81. doi: 10.1007/s00018-011-0846-8. PMID: 21997384 PMC3276771

[4] Kaplan DH Igyártó BZ Gaspari AA . Early immune events in the induction of allergic contact dermatitis. Nat Rev Immunol. (2012) 12:114–24. doi: 10.1038/nri3150. PMID: 22240625 PMC3578582

[5] Losada-Fernández I San Martín A Moreno-Nombela S Suárez-Cabrera L Valencia L Pérez-Aciego P . *In vitro* skin models for skin sensitisation: challenges and future directions. (2025) 12:173. doi: 10.3390/cosmetics12040173

[6] Wong Lau A Perez Pineda J DeLouise LA . Immunomodulatory effects of nanoparticles on dendritic cells in a model of allergic contact dermatitis: importance of PD-L2 expression. Sci Rep. (2023) 13:15992. doi: 10.1038/s41598-023-42797-5. PMID: 37749142 PMC10520013

[7] Martin SF Esser PR . Innate immune mechanisms in contact dermatitis. Handb Exp Pharmacol. (2022) 268:297–310. doi: 10.1007/164_2021_482. PMID: 34173865

[8] Funch AB Geisler C Bonefeld CM . Allergic contact dermatitis: immunopathology and potential therapeutic strategies. J Clin Med. (2025) 14:7175. doi: 10.3390/jcm14207175. PMID: 41156043 PMC12564274

[9] Yamaguchi HL Yamaguchi Y Peeva E . Role of innate immunity in allergic contact dermatitis: an update. Int J Mol Sci. (2023) 24:12975. doi: 10.3390/ijms241612975. PMID: 37629154 PMC10455292

[10] Li J Xiao C Li C He J . Tissue-resident immune cells: from defining characteristics to roles in diseases. Signal Transduction Targeted Ther. (2025) 10:12. doi: 10.1038/s41392-024-02050-5. PMID: 39820040 PMC11755756

[11] Liu AW Gillis JE Sumpter TL Kaplan DH . Neuroimmune interactions in atopic and allergic contact dermatitis. J Allergy Clin Immunol. (2023) 151:1169–77. doi: 10.1016/j.jaci.2023.03.013. PMID: 37149370 PMC10167546

[12] Menzel M Mraz V Vaher H Geisler C Menné Bonefeld C . Metabolic re-programming of keratinocytes in response to contact allergens. Contact Dermatitis. (2024) 90:235–44. doi: 10.1111/cod.14462. PMID: 37985405

[13] Andersen HH Elberling J Arendt-Nielsen L . High-concentration topical capsaicin may abolish the clinical manifestations of allergic contact dermatitis by effects on induction and elicitation. Med Hypotheses. (2017) 99:53–6. doi: 10.1016/j.mehy.2016.12.012. PMID: 28110699

[14] Pinheiro-Rosa N Torres L Oliveira MA Andrade-Oliveira MF Guimarães MAF Coelho MM . Oral tolerance as antigen-specific immunotherapy. Immunother Adv. (2021) 1:ltab017. doi: 10.1093/immadv/ltab017. PMID: 35919733 PMC9327124

[15] Bellinghausen I Khatri R Saloga J . Current strategies to modulate regulatory T cell activity in allergic inflammation. Front Immunol. (2022) 13:912529. doi: 10.3389/fimmu.2022.912529. PMID: 35720406 PMC9205643

[16] Saito K Kitoh A Hanakawa S Nomura T Miyachi Y Kabashima K . Percutaneous exposure to high-dose hapten induces systemic immunosuppression through the inhibition of dendritic cell migration. J Dermatol Sci. (2016) 81:136–40. doi: 10.1016/j.jdermsci.2015.11.011. PMID: 26642795

[17] Lamiable O Mayer JU Munoz-Erazo L Ronchese F . Dendritic cells in Th2 immune responses and allergic sensitization. Immunol Cell Biol. (2020) 98:807–18. doi: 10.1111/imcb.12387. PMID: 32738152

[18] Wilcox NC Taheri G Halievski K Talbot S Silva JR Ghasemlou N . Interactions between skin-resident dendritic and Langerhans cells and pain-sensing neurons. J Allergy Clin Immunol. (2024) 154:11–9. doi: 10.1016/j.jaci.2024.03.006. PMID: 38492673

[19] Toebak MJ Gibbs S Bruynzeel DP Scheper RJ Rustemeyer T . Dendritic cells: biology of the skin. Contact Dermatitis. (2009) 60:2–20. doi: 10.1111/j.1600-0536.2008.01443.x. PMID: 19125717

[20] Yeung K Mraz V Geisler C Skov L Bonefeld CM . The role of interleukin-1β in the immune response to contact allergens. Contact Dermatitis. (2021) 85:387–97. doi: 10.1111/cod.13955. PMID: 34324721

[21] Van Den Eeckhout B Tavernier J Gerlo S . Interleukin-1 as innate mediator of T cell immunity. Front Immunol. (2020) 11:621931. doi: 10.3389/fimmu.2020.621931. PMID: 33584721 PMC7873566

[22] Cumberbatch M Clelland K Dearman RJ Kimber I . Impact of cutaneous IL-10 on resident epidermal Langerhans' cells and the development of polarized immune responses. J Immunol. (2005) 175:43–50. doi: 10.4049/jimmunol.175.1.43. PMID: 15972630

[23] Stutte S Quast T Gerbitzki N Savinko T Novak N Reifenberger J . Requirement of CCL17 for CCR7- and CXCR4-dependent migration of cutaneous dendritic cells. Proc Natl Acad Sci USA. (2010) 107:8736–41. doi: 10.1073/pnas.0906126107. PMID: 20421491 PMC2889308

[24] Kabashima K Sakata D Nagamachi M Miyachi Y Inaba K Narumiya S . Prostaglandin E2-EP4 signaling initiates skin immune responses by promoting migration and maturation of Langerhans cells. Nat Med. (2003) 9:744–9. doi: 10.1038/nm872. PMID: 12740571

[25] Meloun A León B . Beyond CCR7: dendritic cell migration in type 2 inflammation. Front Immunol. (2025) 16:1558228. doi: 10.3389/fimmu.2025.1558228. PMID: 40093008 PMC11906670

[26] Takayama K Yokozeki H Ghoreishi M Satoh T Katayama I Umeda T . IL-4 inhibits the migration of human Langerhans cells through the downregulation of TNF receptor II expression. J Invest Dermatol. (1999) 113:541–6. doi: 10.1046/j.1523-1747.1999.00629.x. PMID: 10504438

[27] Bechetoille N André V Valladeau J Perrier E Dezutter-Dambuyant C . Mixed Langerhans cell and interstitial/dermal dendritic cell subsets emanating from monocytes in Th2-mediated inflammatory conditions respond differently to proinflammatory stimuli. J Leukoc Biol. (2006) 80:45–58. doi: 10.1189/jlb.0205109. PMID: 16614258

[28] Kushwah R Hu J . Role of dendritic cells in the induction of regulatory T cells. Cell Biosci. (2011) 1:20. doi: 10.1186/2045-3701-1-20 21711933 PMC3125210

[29] Lin YL Liang YC Lee SS Chiang BL . Polysaccharide purified from Ganoderma lucidum induced activation and maturation of human monocyte-derived dendritic cells by the NF-kappaB and p38 mitogen-activated protein kinase pathways. J Leukoc Biol. (2005) 78:533–43. doi: 10.1189/jlb.0804481. PMID: 15894585

[30] Diebold SS Kaisho T Hemmi H Akira S Reis e Sousa C . Innate antiviral responses by means of TLR7-mediated recognition of single-stranded RNA. Science. (2004) 303:1529–31. doi: 10.1126/science.1093616. PMID: 14976261

[31] Heil F Hemmi H Hochrein H Ampenberger F Kirschning C Akira S . Species-specific recognition of single-stranded RNA via toll-like receptor 7 and 8. Science. (2004) 303:1526–9. doi: 10.1126/science.1093620. PMID: 14976262

[32] Zepeda-Cervantes J Ramírez-Jarquín JO Vaca L . Interaction between virus-like particles (VLPs) and pattern recognition receptors (PRRs) from dendritic cells (DCs): toward better engineering of VLPs. Front Immunol. (2020) 11:1100. doi: 10.3389/fimmu.2020.01100. PMID: 32582186 PMC7297083

[33] Brand P Plochmann S Valk E Zahn S Saloga J Knop J . Activation and translocation of p38 mitogen-activated protein kinase after stimulation of monocytes with contact sensitizers. J Invest Dermatol. (2002) 119:99–106. doi: 10.1046/j.1523-1747.2002.01791.x. PMID: 12164931

[34] Harris-Tryon TA Grice EA . Microbiota and maintenance of skin barrier function. Science. (2022) 376:940–5. doi: 10.1126/science.abo0693. PMID: 35617415

[35] Bonefeld CM Geisler C Gimenéz-Arnau E Lepoittevin JP Uter W Johansen JD . Immunological, chemical and clinical aspects of exposure to mixtures of contact allergens. Contact Dermatitis. (2017) 77:133–42. doi: 10.1111/cod.12847. PMID: 28677261

[36] Sakamoto E Katahira Y Mizoguchi I Watanabe A Furusaka Y Sekine A . Chemical- and drug-induced allergic, inflammatory, and autoimmune diseases via haptenation. Biol (Basel). (2023) 12:123. doi: 10.3390/biology12010123. PMID: 36671815 PMC9855847

[37] Dibo N Liu X Chang Y Huang S Wu X . Pattern recognition receptor signaling and innate immune responses to schistosome infection. Front Cell Infect Microbiol. (2022) 12:1040270. doi: 10.3389/fcimb.2022.1040270. PMID: 36339337 PMC9633954

[38] Magrone T Russo MA Jirillo E . Impact of heavy metals on host cells: special focus on nickel-mediated pathologies and novel interventional approaches. Endocr Metab Immune Disord Drug Targets. (2020) 20:1041–58. doi: 10.2174/1871530319666191129120253. PMID: 31782370

[39] Bechara R Antonios D Azouri H Pallardy M . Nickel sulfate promotes IL-17A producing CD4+ T cells by an IL-23-dependent mechanism regulated by TLR4 and Jak-STAT pathways. J Invest Dermatol. (2017) 137:2140–8. doi: 10.1016/j.jid.2017.05.025. PMID: 28634033

[40] Sebastião AI Ferreira I Brites G Silva A Neves BM Teresa Cruz M . NLRP3 inflammasome and allergic contact dermatitis: a connection to demystify. Pharmaceutics. (2020) 12:867. doi: 10.3390/pharmaceutics12090867 PMC7560080 32933004

[41] Ihim SA Abubakar SD Zian Z Sasaki T Saffarioun M Maleknia S . Interleukin-18 cytokine in immunity, inflammation, and autoimmunity: biological role in induction, regulation, and treatment. Front Immunol. (2022) 13:919973. doi: 10.3389/fimmu.2022.919973. PMID: 36032110 PMC9410767

[42] Papale A Kummer E Galbiati V Marinovich M Galli CL Corsini E . Understanding chemical allergen potency: role of NLRP12 and Blimp-1 in the induction of IL-18 in human keratinocytes. Arch Toxicol. (2017) 91:1783–94. doi: 10.1007/s00204-016-1806-8. PMID: 27585668

[43] Galbiati V Cornaghi L Papale A Donetti E Marinovich M Corsini E . Study on the inflammasome nlrp3 and blimp-1/nlrp12 after keratinocyte exposure to contact allergens. Toxicol Lett. (2019) 313:130–6. doi: 10.1016/j.toxlet.2019.07.003. PMID: 31276767

[44] Kostarnoy AV Gancheva PG Lepenies B Tukhvatulin AI Dzharullaeva AS Polyakov NB . Receptor Mincle promotes skin allergies and is capable of recognizing cholesterol sulfate. Proc Natl Acad Sci USA. (2017) 114:E2758–e2765. doi: 10.1073/pnas.1611665114. PMID: 28292894 PMC5380039

[45] Kim JH Hu Y Yongqing T Kim J Hughes VA Le Nours J . CD1a on Langerhans cells controls inflammatory skin disease. Nat Immunol. (2016) 17:1159–66. doi: 10.1038/ni.3523. PMID: 27548435 PMC5791155

[46] Natsuaki Y Egawa G Nakamizo S Ono S Hanakawa S Okada T . Perivascular leukocyte clusters are essential for efficient activation of effector T cells in the skin. Nat Immunol. (2014) 15:1064–9. doi: 10.1038/ni.2992. PMID: 25240383

[47] Suwanpradid J Shih M Pontius L Yang B Birukova A Guttman-Yasky E . Arginase1 deficiency in monocytes/macrophages up-regulates iNOS to promote cutaneous contact hypersensitivity. (2017) 199:1827. doi: 10.4049/jimmunol.1700739 PMC5568483 28747341

[48] Liu Y Yin M Mao X Wu S Wei S Heng S . Defining cell type-specific immune responses in a mouse model of allergic contact dermatitis by single-cell transcriptomics. Elife. (2024) 13:RP94698. doi: 10.7554/elife.94698. PMID: 39213029 PMC11364439

[49] Mantovani A Cassatella MA Costantini C Jaillon S . Neutrophils in the activation and regulation of innate and adaptive immunity. Nat Rev Immunol. (2011) 11:519–31. doi: 10.1038/nri3024. PMID: 21785456

[50] Engeman T Gorbachev AV Kish DD Fairchild RL . The intensity of neutrophil infiltration controls the number of antigen-primed CD8 T cells recruited into cutaneous antigen challenge sites. J Leukoc Biol. (2004) 76:941–50. doi: 10.1189/jlb.0304193. PMID: 15328335

[51] Brailey PM Lebrusant-Fernandez M Barral PJTFJ . NKT cells and the regulation of intestinal immunity: a two‐way street. (2020) 287:1686–99. doi: 10.1111/febs.15238 32022989

[52] Zhao L Yang X . Cross Talk Between Natural Killer T and Dendritic Cells and Its Impact on T Cell Responses in Infections. Front Immunol. (2022) 13:837767. doi: 10.3389/fimmu.2022.837767 PMC8850912 35185930

[53] Kumagai K Horikawa T Shigematsu H Matsubara R Kitaura K Eguchi T . Possible immune regulation of natural killer T cells in a murine model of metal ion-induced allergic contact dermatitis. Int J Mol Sci. (2016) 17:87. doi: 10.3390/ijms17010087. PMID: 26771600 PMC4730330

[54] Suwanpradid J Holcomb ZE MacLeod AS . Emerging skin T-cell functions in response to environmental insults. J Invest Dermatol. (2017) 137:288–94. doi: 10.1016/j.jid.2016.08.013. PMID: 27784595 PMC5552043

[55] Hsu U-H Chiang B-L . γδ T cells and allergic diseases. immunology. (2023) 65:172–82. doi: 10.1007/s12016-023-08966-0. PMID: 37395986

[56] Chen Y-L Hardman CS Yadava K Ogg G . Innate lymphocyte mechanisms in skin diseases. Aroi. (2020) 38:171–202. doi: 10.1146/annurev-immunol-082919-093554. PMID: 32340577

[57] Jiménez M Cervantes-García D Córdova-Dávalos LE Pérez-Rodríguez MJ Gonzalez-Espinosa C Salinas E . Responses of mast cells to pathogens: beneficial and detrimental roles. Front Immunol. (2021) 12:685865. doi: 10.3389/fimmu.2021.685865 34211473 PMC8240065

[58] Zhang Z Kurashima Y . Two sides of the coin: mast cells as a key regulator of allergy and acute/chronic inflammation. Cells. (2021) 10:1615. doi: 10.3390/cells10071615. PMID: 34203383 PMC8308013

[59] Carbone T Nasorri F Pennino D Eyerich K Foerster S Cifaldi L . CD56highCD16-CD62L- NK cells accumulate in allergic contact dermatitis and contribute to the expression of allergic responses. J Immunol. (2010) 184:1102–10. doi: 10.4049/jimmunol.0902518. PMID: 20008290

[60] Schuster IS Andoniou CE Degli-Esposti M . Tissue‐resident memory NK cells: homing in on local effectors and regulators. (2024) 323:54–60. doi: 10.1111/imr.13332 PMC11102295 38568046

[61] Perera Molligoda Arachchige AS . Human NK cells: from development to effector functions. Innate Immun. (2021) 27:212–29. doi: 10.1177/17534259211001512. PMID: 33761782 PMC8054151

[62] Paust S von Andrian UH . Natural killer cell memory. Nat Immunol. (2011) 12:500–8. doi: 10.1038/ni.2032. PMID: 21739673

[63] Vocanson M Hennino A Rozières A Poyet G Nicolas JF . Effector and regulatory mechanisms in allergic contact dermatitis. Allergy. (2009) 64:1699–714. doi: 10.1111/j.1398-9995.2009.02082.x. PMID: 19839974

[64] Shah K Al-Haidari A Sun J Kazi JU . T cell receptor (TCR) signaling in health and disease. Signal Transduct Target Ther. (2021). 6:412. doi: 10.1038/s41392-021-00823-w 34897277 PMC8666445

[65] Saint-Mezard P Berard F Dubois B Kaiserlian D Nicolas JF . The role of CD4+ and CD8+ T cells in contact hypersensitivity and allergic contact dermatitis. Eur J Dermatol. (2004) 14:131–8. doi: 10.1201/b14248-37 15246935

[66] Liu X Yu P Xu Y Wang Y Chen J Tang F . Metformin induces tolerogenicity of dendritic cells by promoting metabolic reprogramming. Cell Mol Life Sci. (2023) 80:283. doi: 10.1007/s00018-023-04932-3. PMID: 37688662 PMC10492886

[67] Palomares O Elewaut D Irving PM Jaumont X Tassinari P . Regulatory T cells and immunoglobulin E: a new therapeutic link for autoimmunity? Allergy. (2022) 77:3293–308. doi: 10.1111/all.15449. PMID: 35852798

[68] Alcaide P King SL Dimitroff CJ Lim YC Fuhlbrigge RC Luscinskas FW . The 130-kDa glycoform of CD43 functions as an E-selectin ligand for activated Th1 cells *in vitro* and in delayed-type hypersensitivity reactions *in vivo*. J Invest Dermatol. (2007) 127:1964–72. doi: 10.1038/sj.jid.5700805. PMID: 17392823

[69] Matsumoto M Shigeta A Furukawa Y Tanaka T Miyasaka M Hirata T . CD43 collaborates with P-selectin glycoprotein ligand-1 to mediate E-selectin-dependent T cell migration into inflamed skin. J Immunol. (2007) 178:2499–506. doi: 10.4049/jimmunol.178.4.2499. PMID: 17277158

[70] Azeem M Kader H Kerstan A Hetta HF Serfling E Goebeler M . Intricate relationship between adaptive and innate immune system in allergic contact dermatitis. Yale J Biol Med. (2020) 93:699–709. 33380932 PMC7757059

[71] Yoshizawa T Kumagai K Matsubara R Nasu K Kitaura K Suzuki M . Characterization of metal-specific T-cells in inflamed oral mucosa in a novel murine model of chromium-induced allergic contact dermatitis. Int J Mol Sci. (2023) 24:2807. doi: 10.3390/ijms24032807. PMID: 36769119 PMC9917800

[72] Sadrolvaezin A Pezhman A Zare I Nasab SZ Chamani S Naghizadeh A . Systemic allergic contact dermatitis to palladium, platinum, and titanium: mechanisms, clinical manifestations, prevalence, and therapeutic approaches. MedComm (2020). (2023) 4:e386. doi: 10.1002/mco2.386. PMID: 37873514 PMC10590457

[73] Traidl C Sebastiani S Albanesi C Merk HF Puddu P Girolomoni G . Disparate cytotoxic activity of nickel-specific CD8+ and CD4+ T cell subsets against keratinocytes. J Immunol. (2000) 165:3058–64. doi: 10.4049/jimmunol.165.6.3058. PMID: 10975816

[74] Zemelka-Wiacek M . The interaction among effector, regulatory, and Tγδ cells determines the development of allergy or tolerance to chromium. J Clin Med. (2025) 14:1370. doi: 10.3390/jcm14041370. PMID: 40004900 PMC11856200

[75] Du Y Fang Q Zheng SG . Regulatory T cells: concept, classification, phenotype, and biological characteristics. Adv Exp Med Biol. (2021). 1278:1–31. doi: 10.1007/978-981-15-6407-9_1 33523440

[76] Song Y Wang N Chen L Fang L . Tr1 cells as a key regulator for maintaining immune homeostasis in transplantation. Front Immunol. (2021) 12:671579. doi: 10.3389/fimmu.2021.671579. PMID: 33981317 PMC8109434

[77] Porro C Cianciulli A Panaro MA . The regulatory role of IL-10 in neurodegenerative diseases. Biomolecules. (2020) 10:1017. doi: 10.3390/biom10071017. PMID: 32659950 PMC7407888

[78] Ferguson TA Dube P Griffith TS . Regulation of contact hypersensitivity by interleukin 10. J Exp Med. (1994) 179:1597–604. doi: 10.1084/jem.179.5.1597. PMID: 8163939 PMC2191501

[79] Enk AH Katz SI . Identification and induction of keratinocyte-derived IL-10. J Immunol. (1992) 149:92–5. doi: 10.4049/jimmunol.149.1.92 1607665

[80] Nagata K Nishiyama C . IL-10 in mast cell-mediated immune responses: anti-inflammatory and proinflammatory roles. Int J Mol Sci. (2021) 22:4972. doi: 10.3390/ijms22094972. PMID: 34067047 PMC8124430

[81] Kan AKC Tang WT Li PH . Helper T cell subsets: development, function and clinical role in hypersensitivity reactions in the modern perspective. Heliyon. (2024) 10:e30553. doi: 10.1016/j.heliyon.2024.e30553. PMID: 38726130 PMC11079302

[82] Yamagiwa S Gray JD Hashimoto S Horwitz DA . A role for TGF-beta in the generation and expansion of CD4+CD25+ regulatory T cells from human peripheral blood. J Immunol. (2001) 166:7282–9. doi: 10.4049/jimmunol.166.12.7282. PMID: 11390478

[83] Wang J Zhao X Wan YY . Intricacies of TGF-β signaling in Treg and Th17 cell biology. Cell Mol Immunol. (2023) 20:1002–22. doi: 10.1038/s41423-023-01036-7. PMID: 37217798 PMC10468540

[84] Cederbom L Hall H Ivars F . CD4+CD25+ regulatory T cells down-regulate co-stimulatory molecules on antigen-presenting cells. Eur J Immunol. (2000) 30:1538–43. doi: 10.1002/1521-4141(200006)30:6<1538::aid-immu1538>3.0.co;2-x 10898488

[85] Guglielmo A Borghi A Zengarini C Piraccini BM Corazza M Pileri A . OX40-OX40L axis in cutaneous T-cell lymphomas: pathogenic, prognostic, and potential therapeutic perspectives. Biomolecules. (2025) 15:715. doi: 10.20944/preprints202504.1688.v1 PMC12109069 40427608

[86] Bouaziz JD Yanaba K Tedder TF . Regulatory B cells as inhibitors of immune responses and inflammation. Immunol Rev. (2008) 224:201–14. doi: 10.1111/j.1600-065x.2008.00661.x. PMID: 18759928

[87] Yanaba K Bouaziz JD Haas KM Poe JC Fujimoto M Tedder TF . A regulatory B cell subset with a unique CD1dhiCD5+ phenotype controls T cell-dependent inflammatory responses. Immunity. (2008) 28:639–50. doi: 10.4049/jimmunol.182.supp.97.1 18482568

[88] Bikle DD . Vitamin D regulation of immune function. Curr Osteoporos Rep. (2022) 20:186–93. doi: 10.1016/b978-0-12-386960-9.00001-0. PMID: 35507293 PMC9065668

[89] Yuk JM Shin DM Lee HM Yang CS Jin HS Kim KK . Vitamin D3 induces autophagy in human monocytes/macrophages via cathelicidin. Cell Host Microbe. (2009) 6:231–43. doi: 10.1016/j.chom.2009.08.004. PMID: 19748465

[90] Peroni DG Piacentini GL Cametti E Chinellato I Boner AL . Correlation between serum 25-hydroxyvitamin D levels and severity of atopic dermatitis in children. Br J Dermatol. (2011) 164:1078–82. doi: 10.1111/j.1365-2133.2010.10147.x. PMID: 21087229

[91] Malley R Muller H Norval M Woods G . Vitamin D3 deficiency enhances contact hypersensitivity in male but not in female mice. (2009) 255:33–40. doi: 10.1016/j.cellimm.2008.09.004 19012883

[92] Benson AA Toh JA Vernon N Jariwala SP . The role of vitamin D in the immunopathogenesis of allergic skin diseases. Allergy. (2012) 67:296–302. doi: 10.1111/j.1398-9995.2011.02755.x. PMID: 22171613

[93] Hyppönen E Berry DJ Wjst M Power C . Serum 25-hydroxyvitamin D and IgE - a significant but nonlinear relationship. Allergy. (2009) 64:613–20. doi: 10.1111/j.1398-9995.2008.01865.x 19154546

